# Analysis of cancer perception by elderly people

**DOI:** 10.1590/S1679-45082018AO4155

**Published:** 2018-06-15

**Authors:** Isaac Felipe Leite Braz, Raquel Andresa Duarte Gomes, Mariele Silva de Azevedo, Francisco das Chagas Marison Alves, Danilo Silveira Seabra, Francisco Pignataro Lima, Joabe dos Santos Pereira

**Affiliations:** 1 Universidade Federal do Rio Grande do Norte, Natal, RN, Brazil.; 2 Universidade Estadual da Paraíba, Campina Grande, PB, Brazil.

**Keywords:** Neoplasms, Aged, Perception, Surveys and questionnaires, Information, Neoplasias, Idoso, Percepção, Inquéritos e questionários, Informação

## Abstract

**Objective:**

To evaluate the perception of elderly population about cancer, correlating it with the clinical variables sex, age and past history of cancer.

**Methods:**

The sample was composed of 300 individuals, 174 (58%) women. A questionnaire containing ten questions and based on the Health Information National Trends Survey was used. For statistical analysis, a p value <0.05 was considered significant.

**Results:**

Individuals aged 80 years and older were more likely to believe that regular tests can identify cancer in early stages, compared to elderly aged under 80 years (OR: 0.103; CI95%: 0.021-0.499; p=0.005). Elderly subjects with positive history of cancer were more likely to believe that few people survive cancer, compared to those who never had the disease (OR: 0.379; CI95%: 0.167-0.858; p=0.02). All patients with a positive history of cancer believed that early-detected cancer can be cured.

**Conclusion:**

Aged individuals with ≥80 years or older believed in regular exams as a form of early detection of cancer, probably due to the greater frequency of medical instructions. Subjects who had cancer believed that few people survive the disease, perhaps because of the negative experiences they have experienced. Considering the greater presence of fatalistic perceptions, this group constitutes a potential target for educational approaches about cancer.

## RESUMO

**Objetivo:**

Analisar a percepção do câncer pela população idosa, correlacionando os dados obtidos com as variáveis clínicas de sexo, idade e histórico pessoal de câncer.

**Métodos:**

A amostra foi composta por 300 idosos, sendo 174 (58%) do sexo feminino. Foi aplicado um questionário de dez questões baseado no *Health Information National Trends Survey*, com questões relacionadas à percepção do câncer. Para a análise estatística, o valor de p<0,05 foi considerado significativo.

**Resultados:**

Indivíduos com idade acima de 80 anos demonstram chance maior de acreditar que os exames regulares podem identificar o câncer em estágios iniciais, se comparados aos idosos com menos idade (OR: 0,103; IC95%: 0,021-0,499; p=0,005). Os idosos que tiveram câncer exibiram chances maiores de acreditar que poucas pessoas sobrevivem ao câncer, em relação àquelas que nunca tiveram a doença (OR: 0,379; IC95%: 0,167-0,858; p=0,02). Todos os pacientes com histórico positivo de câncer revelaram acreditar que o câncer detectado cedo pode ser curado.

**Conclusão:**

Idosos com ≥80 anos acreditam nos exames regulares como forma de descobrir a doença no início, provavelmente pela maior frequência de orientações médicas. Indivíduos que tiveram câncer acreditam que poucas pessoas sobrevivem à doença, talvez pelas experiências negativas que vivenciaram. Por exibir mais chances de percepções fatalistas, este grupo se configura como alvo em potencial para abordagens educativas acerca do câncer.

## INTRODUCTION

Cancer is defined as a large group of diseases that can affect any part of the body. It presents an uncontrolled growth of abnormal cells that can spread to other tissues.^(^ ^1^ ^)^ It is also a worldwide public health problem – one of every seven deaths in the world is caused by cancer, which supersedes deaths by HIV/AIDS, tuberculosis and malaria combined.^(^ ^2^ ^)^ The World Health Organization (WHO) estimates that, by 2020, there will be 15 million new cases in the world,^(^ ^3^ ^)^ whereas in Brazil, 420 thousand new cases of cancer were estimated for 2016-2017, not including non-melanoma skin cancer (approximately 180 thousand).^(^ ^4^ ^)^

When dealing with a severe chronic disease such as cancer, the subject of death is present from diagnosis to treatment and post-treatment.^(^ ^5^ ^)^ That is why the Terror Management Health Model suggests that patients avoid situations that may be reminiscent of death, especially when the possibility of decreasing the vulnerability to death seems absent.^(^ ^6^ ^)^ Therefore, perceiving cancer as a death sentence may hinder early diagnosis, adherence to screening programs, and cancer treatment.^(^ ^7^ ^)^

This is important especially regarding elderly patients, among whom the incidence of cancer increases significantly. As people age, there is an accumulation of cancer-specific risk factors,^(^ ^8^ ^)^ including: cumulative exposure to sun and ionizing radiation;^(^ ^2^ ^)^ contact with alcohol, tobacco and air pollution; poor dietary habits; and exposure to infections, all of which are risk factors for a variety of cancers. The most prevalent types of cancer worldwide are: skin, lung, colorectal, prostate,^(^ ^8^ ^)^ and breast.^(^ ^9^ ^)^

In addition to the accumulation of risk factors, the immune system is also compromised with old age and it is therefore less efficient against cancer. Elderly patients present a decreased T cell repertoire in comparison to the clonal variety found in young individuals, which reduces one's ability to react to infections. Senescent T cells do not express costimulatory molecules, such as CD27 and CD28, which are important in the interaction with B-lymphocytes and other antigen-presenting cells for the production of antibodies, and activation and long-term maintenance of T-cells.^(^ ^10^ ^)^ Considering the facts above, expectations for 2030 are that 70% of all cancer cases in the United States will occur in the elderly population.^(^ ^11^ ^)^

Considering that elderly individuals have the highest risk of developing cancer, this population must count on reliable and accurate information to prevent the disease. It is thus paramount that we understand how cancer is perceived by elderly patients. By understanding that, we can predict health-related behaviors and therefore create strategies for health education and better cancer prevention in this specific group.

## OBJECTIVE

To analyze cancer perception in the elderly population, correlating data obtained from the variables sex, age, and past history of cancer.

## METHODS

This research was approved by the Research Ethics Committee of studies conducted in human subjects of *Hospital Universitário Onofre Lopes* - *Universidade Federal do Rio Grande do Norte*, under protocol number 327,967, CAAE: 17263913.0.0000.5292, and was carried out in accordance with resolution 466/12 of the National Health Council and the Declaration of Helsinki. All participants were fully informed and consented to participating in the study and signed an informed consent form.

To calculate the sample, we considered the population of elderly individuals living in the city where the study was conducted 84,323 inhabitants, according to the last census.^(^ ^12^ ^)^ We established the sampling error at 5%, confidence interval at 95%, and homogeneity at 80%, which yielded a recommended sample size of 246 individuals, which was rounded up to 300. The study includes individuals aged over 60 years, admitted to the *Hospital Universitário Onofre Lopes*, in the city of Natal, RN, Brazil, between November 2013 and September 2015, and were randomly selected for an interview.

Initially, the patients were approached by the researchers and received information about the study. Those who agreed to participate signed an Informed Consent Form. After that, we applied a questionnaire with ten questions about cancer perception (Annex 1) based on the Health Information National Trends Survey (HINTS - https://hints.cancer.gov/docs/Instruments/HINTS_4_Cycle_2_English.pdf). The participants were asked how many people they believed usually survive cancer five years after the diagnosis, and about their perception of individual cancer risk. They were also asked if they agreed with some statements about the diversity of recommendations about prevention, the possibility of reducing the risk of cancer, causality, screening, and pessimism.

This research instrument was originally developed from a National Cancer Institute initiative to evaluate the standards of health and cancer information use, and how cancer risks are evaluated by the population, so that researchers could have the means to investigate new theories about health communication.^(^ ^13^ ^)^ Health Information National Trends Survey is currently considered the main data source about cancer related communication in the United States, and has been adapted to and used in research studies in other countries, including in Brazil.^(^ ^14^ ^)^

### Statistical analysis

The statistical analysis was done using the software Statistical Package for Social Sciences (SPSS, Chicago, IL, USA), version 22.0 for Windows, in a descriptive manner and with a multivariate logistic regression model. Obtained data were stratified for sex, age, and history of cancer. Statistical significance was established at p<0.05.

## RESULTS

The total sample included 300 elderly patients, 174 of whom were female (58%). Of those interviewed, 194 (64.6%) were aged 60-69 years, 89 (29.7%) were 70-79 years, and only 17 (5.7%) were 80-89 years. Mean age of population was 67.82 years (minimum 60 years and maximum 88 years). Of all the participants, 29 (9.7%) had a past history of cancer, and the most common types were: eight cases of breast cancers (2.7%), seven cases of prostate cancer (2.3%), and three cases of non-melanoma skin cancer (1%).

In the evaluation of cancer perception, participants were asked ten questions, which gave us a total of 2,723 answers – 1,124 (41.3%) were considered pessimistic and 1,599 (58.7%) were considered optimistic. That showed the predominance of a positive view regarding cancer in the sample (data not shown). Some individuals decided not to answer some questions (277 questions were left unanswered).

Through multivariate logistic regression, we analysed associations of cancer perception and sex, age, and past history of cancer of patients (Table 1). No relevant differences were found in this study between the male and female population regarding cancer perception.

**Table 1:** Associations of perception of cancer and sex, age and past history

	Sex		Age			History of cancer	
	Male OR (95%CI; p value)	Female	60-69 OR (95%CI; p value)	70-79 OR (95%CI; p value)	80-89	No OR (95%CI; p value)	Yes
Few people survive cancer	0.768 (0.449-1.314; p=0.335)	Reference	1.868 (0.492-7.096; p=0.359)	2.543 (0.642-10.069; p=0.184)	Reference	0.379 (0.167-0.858; p=0.02)	Reference
Many recommendations regarding cancer prevention	0.845 (0.475-1.502; p=0.566)	Reference	4.022 (0.507-31.929; p=0.188)	3.403 (0.408-28.344; p=0.258)	Reference	1.227 (0.436-3.453; p=0.698)	Reference
It is difficult to reduce the chance of developing cancer	1.391 (0.848-2.280; p=0.191)	Reference	0.498 (0.147-1.692; p=0.264)	0.499 (0.141-1.767; p=0.281)	Reference	1.979 (0.893-4.384; p=0.093)	Reference
Everything causes cancer	0.918 (0.551-1.529; p=0.743)	Reference	1.121 (0.361-3.481; p=0.843)	0.704 (0.217-2.285; p=0.559)	Reference	0.898 (0.385-2.097; p=0.804)	Reference
Early detected cancer can be cured	0.392 (0.152-1.014; p=0.054)	Reference	1.208 (0.145-10.077; p=0.861)	0.998 (0.110-9.085; p=0.999)	Reference	*	Reference
I can suspect I have cancer before diagnosis	0.704 (0.419-1.183; p=0.185)	Reference	2.179 (0.759-6.251; p=0.148)	1.723 (0.573-5.181; p=0.333)	Reference	1.524 (0.681-3.408; p=0.305)	Reference
Regular exams can identify cancer in an initial phase	0.622 (0.175-2.209; p=0.462)	Reference	0.103 (0.021-0.499; p=0.005)	0.194 (0.038-1.002; p=0.05)	Reference	1.662 (0.192-14.356; p=0.644)	Reference
Cancer is primarily caused by behavior	0.816 (0.469-1.418; p=0.47)	Reference	0.880 (0.281-2.755; p=0.827)	0.577 (0.171-1.949; p=0.376)	Reference	0.653 (0.283-1.510; p=0.319)	Reference
When I think about cancer, I think about death	0.959 (0.591-1.556; p=0.864)	Reference	0.925 (0.329-2.604; p=0.883)	1.277 (0.434-3.762; p=0.657)	Reference	0.451 (0.203-1.002; p=0.05)	Reference
I will very likely develop cancer in the future	0.781 (0.397-1.537; p=0.475)	Reference	0.846 (0.200-3.577; p=0.820)	1.528 (0.333-7.015; p=0.586)	Reference	2.105 (0.772-5.742; p=0.146)	Reference

* It was not possible to carry out the test, since no patient with a positive history of cancer disagreed.

OR: odds ratio; 95%CI: 95% confidence interval.

In relation to age, individuals aged over 80 years showed a statistically higher chance of believing that regular examination can detected cancer in its early stages in comparison to the age group 60–69 years (odds ratio – OR: 0.103; 95% confidence interval – CI 95%: 0.021-0.499; p = 0.005).

With regards to history of cancer, the individuals who said they had previously been diagnosed with cancer showed a higher statistical chance of believing that few people survive cancer, in comparison to those without a history of the disease (OR: 0.379; 95%CI: 0.167-0.858; p=0.02). No participant with a history of cancer disagreed with the statement that cancer can be cured when detected early.

Other associations between the studied variables did not yield statistically significant results.

## DISCUSSION

In general, the elderly population is skeptical of the effectiveness of secondary cancer prevention.^(^ ^15^ ^)^ However, when we stratified this group into categories, certain singularities stood out, which suggests some heterogeneity within this population, especially regarding the perception of cancer screening.

When the interviewed population was stratified into age groups, we found that the subgroup ≥80 years presented the highest chance of believing that regular examination can detect cancer early on. An American study about colorectal cancer screening in elderly patients^(^ ^16^ ^)^ detected that individuals between 65 and 74 years had the lowest percentage in terms of regularly visiting a doctor, which is associated to the fact that they were also not up to date regarding colorectal cancer screening. In light of that finding, a possible explanation for the results of our study would be that individuals aged ≥80 years go to the physician more frequently, and thus have more frequent contact with medical professionals who highlight the importance of screening than the younger population. However, further studies are necessary to confirm this hypothesis.

Regarding history of cancer, elderly with a history of cancer is more likely to believe that few people survive the disease. Considering the significant increase we have seen in the survival rate of several types of cancer,^(^ ^17^ ^–^ ^19^ ^)^ including in Brazil,^(^ ^20^ ^)^ we would expect that those who have survived it would be more optimistic and believe that a considerable number of people can live many years after treatment. Nevertheless, our results point in the opposite direction.

This apparent contradiction is better understood when we consider the Dual Processing Theory,^(^ ^19^ ^,^ ^21^ ^)^ which states that human beings are able to react with different intensities to memories of positive and negative past experiences because the brain processes these situations differently. A study from the United Kingdom^(^ ^19^ ^)^ applied these concepts in the context of cancer, and obtained pessimistic answers more quickly and with a higher emotional charge in comparison to optimistic answers, which were given second and with a much lower emotional charge. Another important point described by the Dual Processing Theory is that a mental picture that we can easily put together is interpreted by the brain as a frequently occurring event.^(^ ^19^ ^)^

Therefore, we can suggest that once diagnosed with cancer and thus immersed in healthcare providing environments, elderly patients will be in contact with other patients' positive and negative experiences. Due to dual processing, negative experiences are more powerful and therefore more vividly stored in the mind. Consequently, these negative experiences are perceived to be more frequent, which is why there is this perception that most cancer patients have a low survival rate.

It is noteworthy that, even though the Dual Processing Theory as described above can explain pessimistic perception after diagnosis of cancer, what we present here is a cross-sectional study, and so it does not show the relation of temporality between the construct of pessimistic perception of cancer and past history of cancer. Therefore, we do not know if the individual had a pessimistic conception even before the diagnosis, or if his pessimistic perception was formed after the diagnosis.

Even though individuals with a history of cancer tend to have pessimistic thoughts about cancer survival, in our study we found that all participants with a history of the disease believe that cancer can be cured when detected early. One possible explanation for this optimistic conception is the increase of mass educational campaigns that spread information about the importance of an early diagnosis. In Brazil, the relevance of health-related programs against cancer has been recognized since 1920,^(^ ^22^ ^)^ to mobilize the society to take part in actively preventing the disease by raising awareness and seeking behavioral changes.^(^ ^23^ ^)^

One study from the United States^(^ ^24^ ^)^ found similar results to ours, and reported that individuals with a past history of cancer have more concerns about having new cancers and have a higher risk of developing different neoplasms,^(^ ^24^ ^,^ ^25^ ^)^ which might motivate them to more actively seek an early diagnosis and methods of prevention.

Knowledge dissemination is important regardless of a past history of cancer. In our study, patients with or without a history of the disease believe that an early cancer diagnosis improves their chances of survival: that was true of 91.0% of the interviewed participants (data not shown) in our study, which is similar to what many other authors observed.^(^ ^24^ ^)^

Regarding the individuals without a history of cancer, our study showed that they tend not to think about death when they think of cancer. However, a study conducted in the United States found different results, observing that patients without a history of cancer avoid medical appointments, which they believe evoke death-related thoughts.^(^ ^26^ ^)^ The study also found that those with a history of cancer tend to not to think of cancer as a death sentence.^(^ ^24^ ^)^

The difference between our results and those obtained in the abovementioned study can be explained by analyzing the prevalence of cancer survivors, which is the number of survivors per 100 thousand diagnosed adults in both countries, in the last 5 years. In Brazil, this prevalence is 720.2, while in the United States, it is 1,892.1, which shows poorer survival of Brazilians than of Americans.^(^ ^27^ ^)^ Among the possible causes are low access to medical resources, insufficient availability of certain public services, a system that cannot keep up with the demand for healthcare, and a shortage of investments in healthcare.^(^ ^28^ ^)^

The American Cancer Society (ACS) stated the survival of patients diagnosed with cancer in one particular country is an important measurement of the efficacy of that country's health system regarding cancer diagnosis, management, and treatment. Another data that suggests a poorer performance of the Brazilian health system can be observed when we compare years of life lost due to cancer per 100 thousand individuals. Brazilians lose 2,117 years, while Americans lose 2,031.^(^ ^27^ ^)^

Thus, considering the challenges cancer patients face when dealing with the Brazilian health system, and the difficulties that are inherent to cancer treatment, which is aggressive, invasive and stressful,^(^ ^5^ ^)^ we can understand the pessimistic view of participants with a history of the disease in comparison to those without a history – the latter have never faced these obstacles and difficulties and therefore tend not to associate cancer with death.

The results of the study provide behavioral data related to health of elderly patients that can aid in the development of education and cancer prevention strategies for this population. Additionally, disseminating appropriate information to the elderly population will reduce the level of concern and anxiety generated by the possibility of developing cancer or by the experience of having the disease.

## CONCLUSION

Elderly patients aged ≥80 years showed a greater chance of believing that regular examinations can detected cancer in its initial stages, which may be related to the higher frequency of medical visits in this age group. Individuals with a previous history of cancer, in turn, show a higher chance of believing that few people can survive the disease, which may be associated to the more vivid intensity of negative memories related to cancer. Furthermore, all interviewees with a previous history of cancer agreed that cancer can be cured when detected early, which is probably due to the many prevention campaigns that stress the importance of an early diagnosis. The conceptions observed in the studied sample are potential targets for educational and communicative approaches that seek better efficacy in cancer prevention.

## Annex 1. Questionnaire about cancer perception*

**Table:**

Age		Have you had cancer?	
Sex M ( ) F ( )		What type?	
**1. In your opinion, how many people with cancer remain alive 5 years after developing the disease?**			
Few	( )	Almost everyone	( )
Half	( )	Refuses to answer	( )
Many	( )	Does not know	( )
**Tell me if you agree, disagree or have no opinion regarding the following statements:**
**2. There are so many different recommendations about preventing cancer, it's hard to know which ones to follow.**				
Agree	( )	Refuses to answer	( )
Disagree	( )	Does not know	( )
**3. There's not much you can do to lower your chances of getting cancer.**			
Agree	( )	Refuses to answer	( )
Disagree	( )	Does not know	( )
**4. It seems like everything causes cancer.**			
Agree	( )	Refuses to answer	( )
Disagree	( )	Does not know	( )
**5. Cancer is a disease that can be cured when detected early.**			
Agree	( )	Refuses to answer	( )
Disagree	( )	Does not know	( )
**6. People can believe to have cancer before being diagnosed.**			
Agree	( )	Refuses to answer	( )
Disagree	( )	Does not know	( )
**7. If I undergo regular cancer screening I can detect the disease at a stage when it is easier to treat it.**			
Agree	( )	Refuses to answer	( )
Disagree	( )	Does not know	( )
**8. Cancer is mainly caused by one's behavior or life style.**			
Agree	( )	Refuses to answer	( )
Disagree	( )	Does not know	( )
**9. When I think about cancer, I automatically think of death.**			
Agree	( )	Refuses to answer	( )
Disagree	( )	Does not know	( )
**10. In your opinion, how likely are you to develop cancer in your lifetime?**			
Very unlikely (0-25%)	( )	Very likely (100%)	( )
Unlikely (25-50%)	( )	Refuses to answer	( )
Neither unlikely nor likely (50-75%)	( )	Does not know	( )
Likely (75-99%)	( )		

* Based on: Health Information National Trends Survey (HINTS - https://hints.cancer.gov/docs/Instruments/HINTS_4_Cycle_2_English.pdf).

## References

[1] World Health Organization (WHO) (2018). Cancer. What is cancer? [Internet].

[2] American Cancer Society (ACS) (2017). Cancer Facts & Figures 2017 [Internet].

[3] World Health Organization (WHO) (2018). Global cancer rates could increase by 50% to 15 million by 2020 [Internet].

[4] Instituto Nacional de Câncer José Alencar Gomes da Silva (INCA) (2015). Estimativa 2016: incidência de câncer no Brasil [Internet].

[5] Borges AD, Silva EF, Toniollo PB, Mazer SM, Valle ER, Santos MA (2006). Percepção da morte pelo paciente oncológico ao longo do desenvolvimento. Psicol Estudo.

[6] Goldenberg JL, Arndt J (2008). The implications of death for health: a terror management health model for behavioral health promotion. Psychol Rev.

[7] Powe BD, Finnie R (2003). Cancer fatalism: the state of the science. Cancer Nurs.

[8] Danaei G, Vander Hoorn S, Lopez AD, Murray CJ, Ezzati M (2005). Comparative Risk Assessment collaborating group (Cancers). Causes of cancer in the world: comparative risk assessment of nine behavioural and environmental risk factors. Lancet.

[9] Miranda TC, Kaliks RA, Jacob W, Giglio AD (2008). Breast cancer in elderly women – perspective of geriatricians. einstein (São Paulo).

[10] Xu W, Larbi A (2017). Markers of T Cell Senescence in Humans. Int J Mol Sci.

[11] Smith BD, Smith GL, Hurria A, Hortobagyi GN, Buchholz TA (2009). Future of cancer incidence in the United States: burdens upon an aging, changing nation. J Clin Oncol.

[12] Instituto Brasileiro de Geografia e Estatística (IBGE) (2010). Censo demográfico 2010 [Internet].

[13] Cantor D, Coa K, Crystal-Mansour S, Davis T, Dipko S, Sigman R (2009). Health Information National Trends Survey (HINTS) 2007 [Internet]. Final Report.

[14] Reddy S, Pimenta DN, Kaplan-Liss E, Guimarães M (2003). Sources of Health Information In Brazil: a perspective from students of the federal university of Rio de Janeiro. Ponto Acesso.

[15] Balducci L (2016). Cancer prevention in the older individual. Semin Oncol Nurs.

[16] Berkowitz Z, Hawkins NA, Peipins LA, White MC, Nadel MR (2008). Beliefs, risk perceptions, and gaps in knowledge as barriers to colorectal cancer screening in older adults. J Am Geriatr Soc.

[17] Coleman MP, Forman D, Bryant H, Butler J, Rachet B, Maringe C, Maringe C, Nur U, Tracey E, Coory M, Hatcher J, McGahan CE, Turner D, Marrett L, Gjerstorff ML, Johannesen TB, Adolfsson J, Lambe M, Lawrence G, Meechan D, Morris EJ, Middleton R, Steward J, Richards MA (2011). ICBP Module 1 Working Group. Cancer survival in Australia, Canada, Denmark, Norway, Sweden, and the UK 1995-2007 (the International Cancer Benchmarking Partnership): an analysis of population-based cancer registry data. Lancet.

[18] DeSantis CE, Lin CC, Mariotto AB, Siegel RL, Stein KD, Kramer JL (2014). Cancer treatment and survivorship statistics, 2014. CA Cancer J Clin.

[19] Robb KA, Simon AE, Miles A, Wardle J (2014). Public perceptions of cancer: a qualitative study of the balance of positive and negative beliefs. BMJ Open.

[20] Silva GA, Gamarra CJ, Girianelli VR, Valente JG (2011). Cancer mortality trends in Brazilian state capitals and other municipalities between 1980 and 2006. Rev Saude Publica.

[21] Slovic P, Finucane ML, Peters E, MacGregor DG (2004). Risk as analysis and risk as feelings: some thoughts about affect, reason, risk, and rationality. Risk Anal.

[22] Teixeira LA, Fonseca CO (2007). De doença desconhecida a problema de saúde pública: o INCA e o controle do Câncer no Brasil.

[23] Costa MC, Teixeira LA (2010). As campanhas educativas contra o câncer. Hist Cienc Saude-Manguinhos.

[24] Kowalkowski MA, Hart SL, Du XL, Baraniuk S, Latini DM (2012). Cancer perceptions: implications from the 2007 Health Information National Trends Survey. J Cancer Surviv.

[25] Teppo L, Salminen E, Pukkala E (2001). Risk of a new primary cancer among patients with lung cancer of different histological types. Eur J Cancer.

[26] Moser RP, Arndt J, Han PK, Waters EA, Amsellem M, Hesse BW (2014). Perceptions of cancer as a death sentence: prevalence and consequences. J Health Psychol.

[27] American Cancer Society (ACS) (2018). International Agency for Research on Cancer. The Cancer Atlas. Compare countries [Internet].

[28] Souza GC, Costa IC (2010). O SUS nos seus 20 anos: reflexões num contexto de mudanças. Saude Soc.

